# More Evidence of Collusion: a New Prophage-Mediated Viral Defense System Encoded by Mycobacteriophage Sbash

**DOI:** 10.1128/mBio.00196-19

**Published:** 2019-03-19

**Authors:** Gabrielle M. Gentile, Katherine S. Wetzel, Rebekah M. Dedrick, Matthew T. Montgomery, Rebecca A. Garlena, Deborah Jacobs-Sera, Graham F. Hatfull

**Affiliations:** a Department of Biological Sciences, University of Pittsburgh, Pittsburgh, Pennsylvania, USA; College of Veterinary Medicine, Cornell University

**Keywords:** bacteriophage, *Mycobacterium*, viral defense

## Abstract

The arms race between bacteria and their bacteriophages profoundly influences microbial evolution. With an estimated 10^23^ phage infections occurring per second, there is strong selection for both bacterial survival and phage coevolution for continued propagation. Many phage resistance systems, including restriction-modification systems, clustered regularly interspaced short palindromic repeat-Cas (CRISPR-Cas) systems, a variety of abortive infection systems, and many others that are not yet mechanistically defined, have been described. Temperate bacteriophages are common and form stable lysogens that are immune to superinfection by the same or closely related phages. However, temperate phages collude with their hosts to confer defense against genomically distinct phages, to the mutual benefit of the bacterial host and the prophage. Prophage-mediated viral systems have been described in *Mycobacterium* phages and *Pseudomonas* phages but are predicted to be widespread throughout the microbial world. Here we describe a new viral defense system in which the mycobacteriophage Sbash prophage colludes with its Mycobacterium smegmatis host to confer highly specific defense against infection by the unrelated mycobacteriophage Crossroads. Sbash genes *30* and *31* are lysogenically expressed and are necessary and sufficient to confer defense against Crossroads but do not defend against any of the closely related phages grouped in subcluster L2. The mapping of Crossroads defense escape mutants shows that genes *132* and *141* are involved in recognition by the Sbash defense system and are proposed to activate a loss in membrane potential mediated by Sbash gp30 and gp31.

## Abstract (precis)

Viral infection is an ongoing challenge to bacterial survival, and there is strong selection for development or acquisition of defense systems that promote survival when bacteria are attacked by bacteriophages. Temperate phages play central roles in these dynamics through lysogenic expression of genes that defend against phage attack, including those unrelated to the prophage. Few prophage-mediated viral defense systems have been characterized, but they are likely widespread both in phage genomes and in the prophages integrated in bacterial chromosomes.

## INTRODUCTION

The bacteriophage population is vast, dynamic, and old (1). The microbial arms race between bacteriophage predators and bacterial prey has promoted evolution of a phage population that is genetically enormously diverse (1), with strong selection for bacterial acquisition of phage resistance mechanisms to defend against viral attack and the need for phage coevolution (2). The phage response to resistance involves both development of antidefense systems (including antirestriction and anticlustered regularly interspaced short palindromic repeat-Cas [CRISPR-Cas] systems) and alteration of host range so that a new spectrum of bacterial strains can be infected (3–6).

Although a plethora of bacterially encoded phage defense systems have been described (7–9), recent evidence suggests that temperate bacteriophages collude extensively with their bacterial hosts to provide defense against viral attack (10, 11); these extend beyond well-known homotypic defenses (i.e., against themselves) such as repressor-mediated superinfection immunity to novel heterotypic defense systems (i.e., against genomically different phages), including some with exquisite specificity (11). Such prophage-mediated heterotypic defense systems may be widespread, but few have been described in detail or are mechanistically understood.

A substantial proportion of bacteriophages are temperate, forming stable lysogens in their bacterial hosts (12, 13). Establishment of lysogeny occurs at relatively high frequencies, especially at high bacterial densities and high multiplicities of infection, and a high proportion of sequenced bacterial genomes carry one or more prophages (14–16). Expression of prophage-carried genes during lysogeny can profoundly influence host physiology, endowing pathogenic properties or additional metabolic pathways (15, 17). However, prophage-encoded viral defense systems are capable of providing survival advantages in multiple environments and may thus be common among temperate phages. These systems have been largely overlooked because of the dearth of well-defined collections of phages that infect common bacterial host strains.

Over 10,000 individual phages have been isolated using Mycobacterium smegmatis mc^2^155 as a host, and over 1,700 have been completely sequenced (https://phagesdb.org) (18). These span a considerable range of genetic diversity and can be assorted into 34 genome types based on shared nucleotide sequences and gene content (29 clusters [A, B, C….Z, AA, AB, AC] and five singletons) (19). There is substantial diversity within the clusters, 13 of which can be divided into subclusters (19). Although over one-half of the different types correspond to temperate phages, prophage-mediated defense systems have been described only for phages grouped in cluster N, which collectively have at least five distinct defense systems (11).

Here we describe a new heterotypic prophage-mediated defense system encoded by the cluster I2 mycobacteriophage Sbash. Two Sbash genes, *30* and *31*, are required for defense, and the system targets the cluster L2 phage Crossroads with remarkable specificity; it does not defend against any other mycobacteriophage tested, including other closely related subcluster L2 phages. Isolation and characterization of defense escape mutants showed that Crossroads genes *132* and *141* are required for identification of Crossroads as the target of defense. We also show that distantly related homologues of the Sbash *30*/*31* defense genes present in *Gordonia* phage CarolAnn also defend against Crossroads when expressed in M. smegmatis.

## RESULTS

### Mycobacteriophage Sbash encodes at least two distinct phage defense systems.

**FIG 1:**
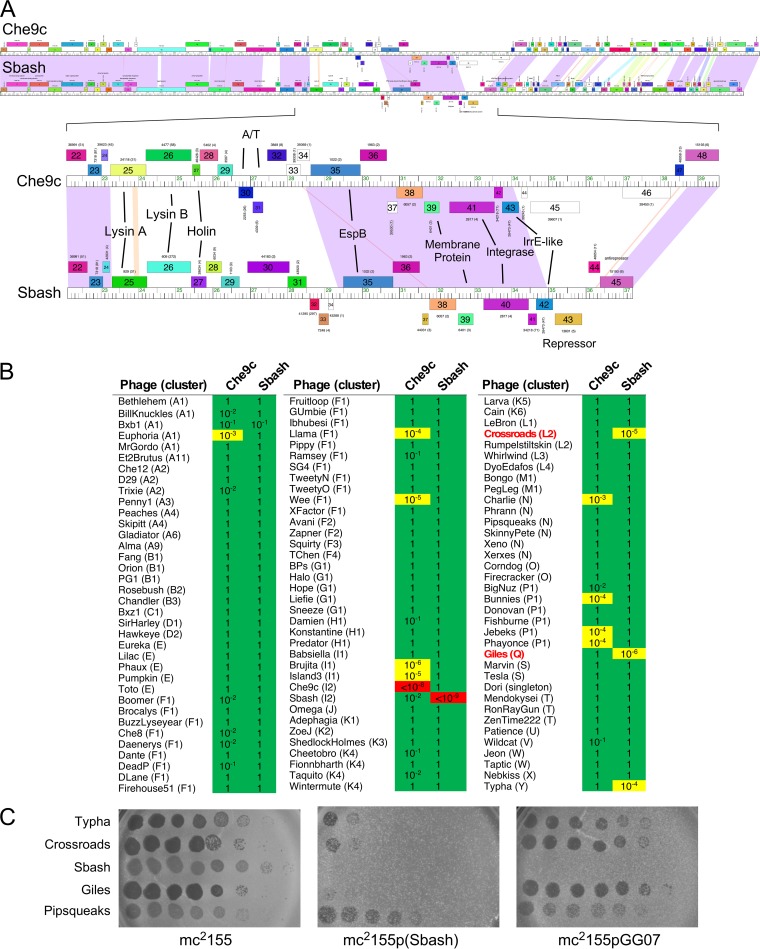
Viral defense systems in mycobacteriophages Sbash and Che9c. (A) The genome maps of phages Che9c and Sbash are shown with shared nucleotide sequence similarity represented as spectrum-colored shading between the genomes, with violet representing the most similar and red the least similar above a BLASTN E value threshold of 10^−4^. Genes are shown as colored boxes above and below genome rulers. An expanded view of the centers of the Che9c and Sbash genomes with putative gene functions indicated is shown at the bottom. Gene numbers are shown within the gene boxes, and the assigned Pham numbers are shown above the boxes, with the number of Pham members indicated in parentheses. (B) Plating efficiencies of mycobacteriophages on lysogens of phages Che9c and Sbash. A total of 108 mycobacteriophages were serially diluted and plated onto lawns of M. smegmatis mc^2^155, mc^2^155(Che9c), and mc^2^155(Sbash). The plating efficiencies on each of the lysogens are shown relative to plating on the nonlysogen. The cluster assignment is shown in parentheses following each phage name, and phages are sorted according to their cluster designation; Giles and Crossroads are highlighted in red type. Plating efficiencies between 1 and 10^−2^, between 10^−3^ and 10^−7^, and at 10^−8^ or below are highlighted in green, yellow, and red, respectively. (C) Sbash-mediated defense against Typha, Crossroads and Giles is not repressor mediated. Ten-fold serial dilutions of phages Typha, Giles, Crossroads, Sbash, and Pipsqueaks were plated onto lawns of mc^2^155, mc^2^155(Sbash), and mc^2^155pGG07, which carries the Sbash repressor (see Fig. 3). Like strain mc^2^155(Sbash), strain mc^2^155pGG07 confers immunity to Sbash but does not inhibit infection by Typha, Crossroads or Giles. Phage Pipsqueaks is a control that is not affected by Sbash defenses. The pGH1000 vector control is shown in Fig. 3.

Mycobacteriophage Sbash was isolated in Durban, South Africa, has a 55,832-bp genome, and is grouped in cluster I (20). Cluster I has only five member phages (https://phagesdb.org): three in subcluster I1 and two—Sbash and Che9c—in subcluster I2 (21). Sbash and Che9c are temperate but heteroimmune (Fig. 1A and B), sharing extensive nucleotide similarity across the structural genes in the left side of the genome and at the extreme right genome ends (Fig. 1A). The central regions have 7 to 8 shared genes (e.g., Sbash *35* to *42*) and several that are present in only one of the two phages (Sbash *28*–*34*; Che9c *28*–*34*) (Fig. 1A). It was shown previously that genes centrally located in cluster N mycobacteriophage genomes are lysogenically expressed and confer defense against other mycobacteriophages (11). We therefore constructed Che9c and Sbash lysogens and tested them for susceptibility to infection by the use of a panel of genomically diverse phages that infect M. smegmatis mc^2^155.

A set of 108 phages were serially diluted and plated onto lawns of M. smegmatis mc^2^155 and lysogens of Sbash and Che9c and the efficiencies of plating (EOP) determined (Fig. 1B). Most of the phages showed little or no difference in EOP, but substantial reductions were observed for several phages on each of the lysogens. These included Crossroads, Giles, and Typha on the Sbash lysogen and phages Euphoria, Llama, Wee, Charlie, Bunnies, Jebeks, and Phayonce on the Che9c lysogen (Fig. 1B); the Sbash and Che9c lysogens showed immunity to themselves but not to each other, and the cluster I phages Brujita and Island3 showed reduced plating on Che9c, probably reflecting homoimmunity. In general, the Sbash and Che9c profiles are nonoverlapping, suggesting the presence of distinct defense systems in the two prophages (Fig. 1B). To determine whether the Sbash defense against Crossroads, Giles, and Typha is repressor mediated, we tested whether these phages can infect a recombinant strain (mc^2^155pGG07) expressing the Sbash repressor (gp43; Fig. 1C). All three phages infect mc^2^155pGG07 efficiently, and the Sbash prophage-mediated defense is thus distinct from repressor-mediated superinfection immunity. Here we further characterized the Sbash defenses; Che9c defenses are to be described elsewhere.

### Identification of Sbash genes conferring viral defense.

**FIG 2:**
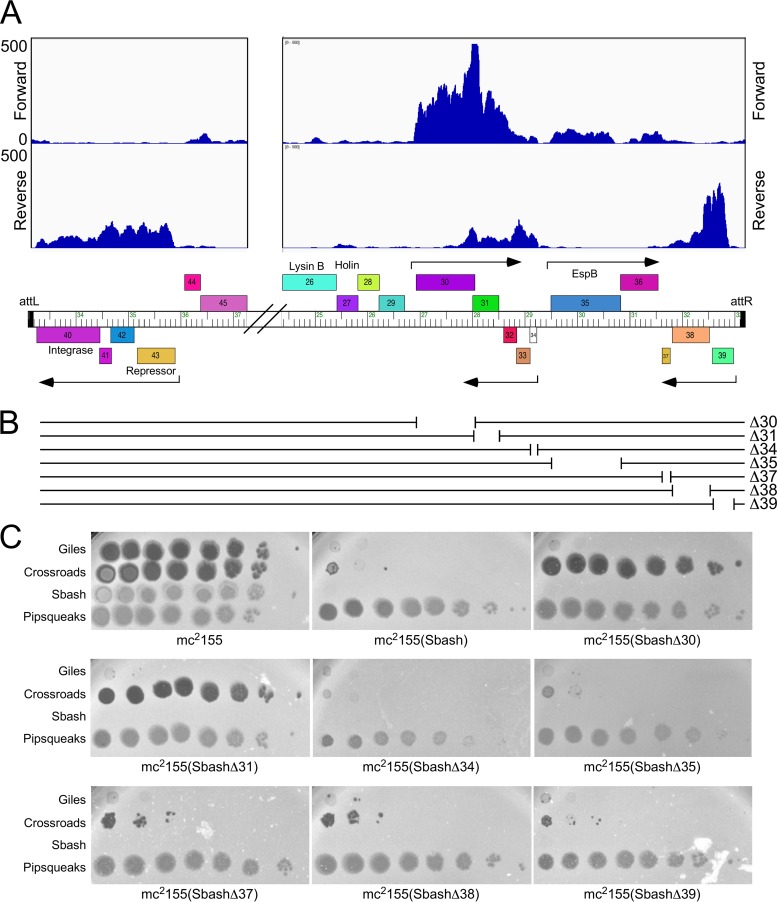
Sbash genes required for viral defense. (A) Lysogenic expression of Sbash prophage genes. RNAseq reads are mapped to the Sbash prophage, shown in its integrated orientation from *attL* to *attR*, with the relevant part of the genome map shown at the bottom. Putative lysogenically expressed operons are represented by arrows. The RNAseq reads are strand specific, and those mapping to forward and reverse DNA orientations are indicated. Results of RNAseq analysis of the whole prophage are shown in Fig. S1. (B) Sbash deletion derivatives. The seven deletion mutants are depicted, showing the region removed in each mutant. Genomes are aligned with the map shown at the top. (C) Plating efficiencies of phages on mutant Sbash lysogens. Ten-fold serial dilutions of phages Giles, Crossroads, Sbash, and Pipsqueaks were plated onto lawns of M. smegmatis mc^2^155 and mc^2^155(Sbash) and of mutant derivatives in which individual Sbash genes were knocked out. Sbash *30* and *31* are both required for defense against Crossroads; none of the deleted genes are required for defense against Giles infection.

To determine which Sbash genes are lysogenically expressed, RNA was isolated from a Sbash lysogen and analyzed by transcriptome sequencing (RNAseq) and the sequence reads were mapped to the Sbash genome (Fig. 2A; see also Fig. S1 in the supplemental material). Expression of five groups of genes was observed near the center of the viral genome, positioned near the ends of the integrated prophage (Fig. 2A). These included the repressor and integrase genes (genes *40* to *43*) adjacent to *attL*, two operons of leftward-transcribed genes (genes *37* to *39* and *32* to *34*), and two rightward-transcribed operons (genes *30*, *31*, and *35* to *36*; Fig. 2A). Most of these genes are of unknown function, with the exceptions being gene *35*, which encodes a homologue of the Esx-A component EspB (22), gene *39*, which codes for a predicted membrane-localized protein, and gene *42*, which codes for an IrrE-like protein (Fig. 2A).

10.1128/mBio.00196-19.1 FIG S1 RNAseq profile of Sbash gene expression in a M. smegmatis mc2155(Sbash) lysogenic strain. Sequence reads were mapped to the prophage, represented here as the viral linear form of the genome. Forward and reverse strands are indicated, and a genome map is shown at the bottom. Download FIG S1, PDF file, 0.2 MB. Copyright © 2019 Gentile et al. 2019 Gentile et al. This content is distributed under the terms of the Creative Commons Attribution 4.0 International license.

To determine which of these genes are responsible for viral defense, we used Bacteriophage Recombineering of Electroporated DNA (BRED) mutagenesis (23) to construct deletion mutants of genes *30*, *31*, *34*, *35*, *37*, *38*, and *39* (Fig. 2B). All of these mutants grow lytically and form stable lysogens in M. smegmatis. Lysogenic strains of the mutants were recovered and tested for phage susceptibility (Fig. 2C). Five of the mutant derivatives (mutants Δ*34*, Δ*35*, Δ*37*, Δ*38*, Δ*39*) had susceptibility profiles similar to that of wild-type Sbash for all the tested phages (Fig. 2C), but both the Δ*30* and Δ*31* mutant lysogens lost defense against Crossroads while maintaining defense against Giles (Fig. 2C). Sbash *30* and *31* thus are required for defense against Crossroads but not for defense against Giles. All of the deletion mutants tested retained defense against Giles, and Sbash genes *32*, *33*, *36*, *41*, and *42* remain plausible candidates for Giles defense genes.

**FIG 3:**
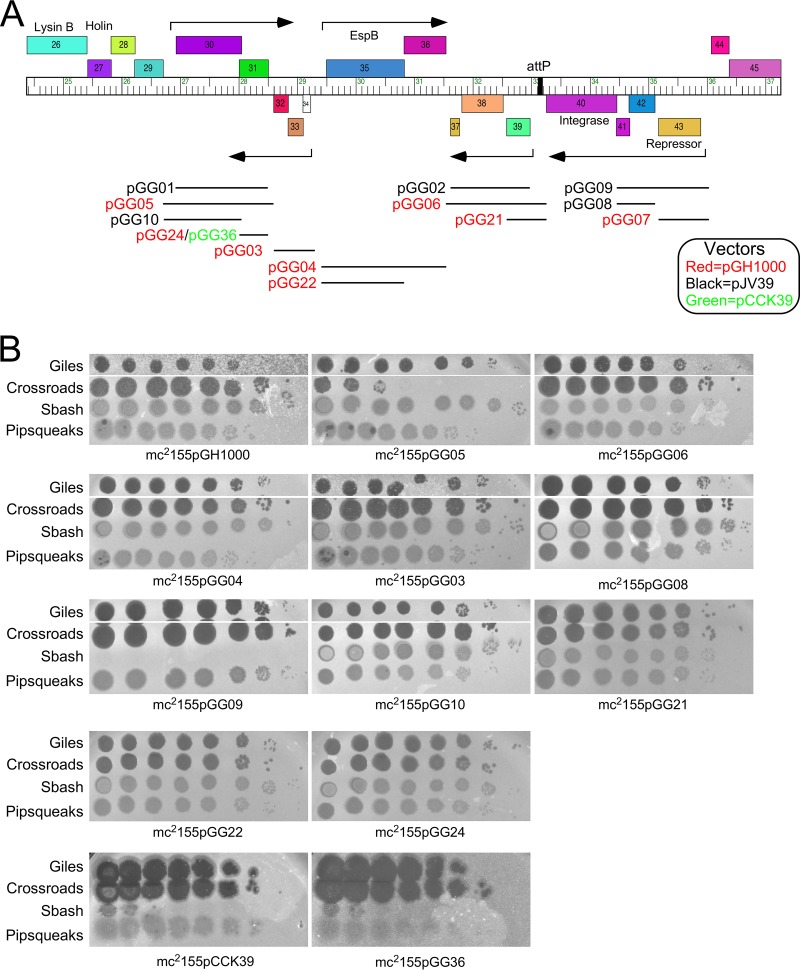
Sbash *30* and *31* are necessary and sufficient to defend against Crossroads. (A) Recombinant clones tested for defense against phage Crossroads and Giles infection. A map of the central portion of the Sbash genome is shown with the insertions in plasmids indicated by horizontal lines and plasmid names. Plasmid vectors are all integration-proficient plasmids based on pGH1000 (red), pJV39 (black), or pCCK39 (green). Phages plate similarly on pJV39 and pGH1000 strains (data not shown). (B) Plating efficiencies of phages Giles, Crossroads, Sbash, and Pipsqueaks on recombinant strains. Ten-fold serial dilutions of each phage were spotted onto lawns of each strain, as indicated. All four phages infected mc^2^155pGH1000 and mc^2^155pJV39 (not shown) vector strains similarly.

To test whether Sbash genes *30* and *31* are sufficient for Crossroads defense, we constructed a series of recombinant plasmids containing subsets of Sbash genes (Fig. 3A). A plasmid carrying Sbash *30* and *31* together with the upstream expression signals (pGG05) conferred a level of Crossroads defense similar to that of a Sbash lysogen, and both gene *30* and gene *31* were required; plasmids pGG10 and pGG24 carrying gene *30* and gene *31* alone, respectively, did not confer defense (Fig. 3B). Thus, Sbash genes *30* and *31* are necessary and sufficient for defense against Crossroads infection; they do not defend against Typha (data not shown). None of the plasmids conferred defense against Giles, perhaps either because noncontiguous genes are required for defense or because the defense genes are not efficiently expressed in the recombinant strains. Although the genetic basis for defense against Giles infection remains unclear, there is clearly a second viral defense system distinct from that corresponding to Sbash *30* and *31*.

### Sbash prophage-mediated defense specificity for phage Crossroads.

In the initial screening for defense patterns (Fig. 1B), none of the other cluster L phages tested (Rumpelstiltskin [L2]; LeBron [L1]; Whirlwind [L3]; DyoEdafos [L4]) were subject to Sbash defense.

**FIG 4:**
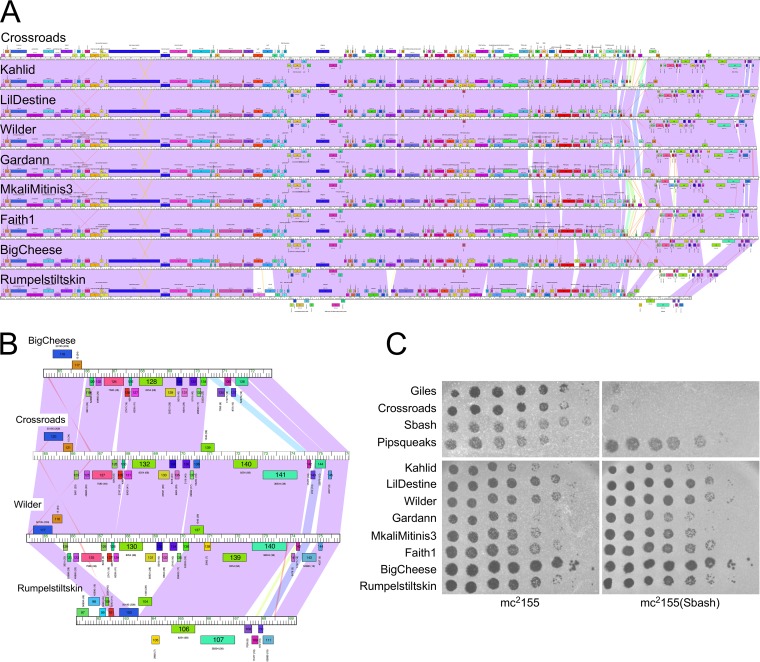
Crossroads is the only subcluster L2 phage targeted by Sbash gp30/gp31 defense. (A) Genome maps of subcluster L2 phages, Crossroads, Kahlid, LilDestine, Wilder, Gardann, MkaliMitinis3, Faith1, BigCheese, and Rumpelstiltskin (top to bottom). Genomes are represented as described for Fig. 1. (B) Expanded view of the right genome ends of phages BigCheese, Crossroads, Wilder, and Rumpelstiltskin. Crossroads and Wilder were very similar in this region, whereas BigCheese and Rumpelstiltskin have 3-kbp to 5-kbp deletions. (C) Plating efficiencies of subcluster L2 phages on mc^2^155 and a Sbash lysogen. Ten-fold serial dilutions of each phage were plated as indicated.

Crossroads and Rumpelstiltskin are closely related to each other, but Rumpelstiltskin has a deletion of ∼5 kbp relative to Crossroads near its right end (Fig. 4A and B). We tested seven additional subcluster L2 phages (Kahlid, LilDestine, Wilder, Gardann, MkaliMitinis3, Faith1, and BigCheese) for infection of a Sbash lysogen to further define the defense specificity (Fig. 4C). Somewhat surprisingly, none of these other subcluster L2 phages had reduced EOP on the Sbash lysogen (Fig. 4C), and the Sbash prophage targeted Crossroads with exquisite specificity.

### Crossroads genes *132* and *141* are involved in Sbash-mediated defense targeting.

**FIG 5:**
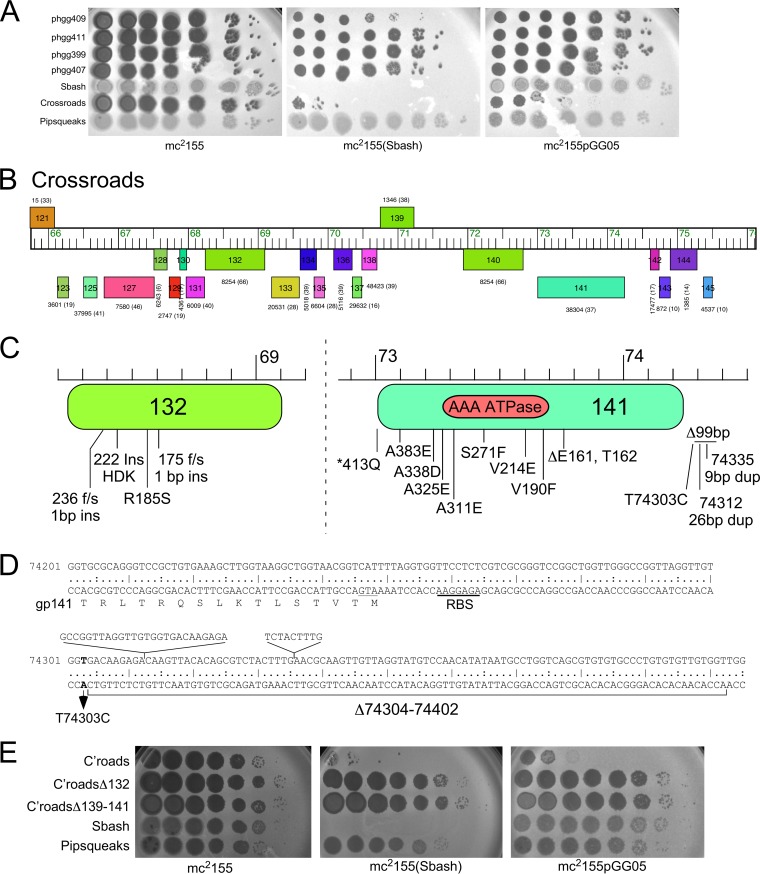
Characterization of Crossroads defense escape mutants. (A) Plating of Crossroads defense escape mutants (DEMs) on host strains. Crossroads mutants were isolated as escapees on lawns of either a Sbash lysogen or a pGG05 recombinant strain, purified, and shown to escape Sbash prophage defense. Ten-fold serial dilutions of DEMs (phgg409, phgg411, phgg399, phgg407) were plated on bacterial lawns as indicated. (B) Organization at the extreme right end of the Crossroads genome showing genes *121* to *145*. The genome is displayed as described for Fig. 1. (C) Mapping of Crossroads DEM mutations. Defense escape mutants of Crossroads were purified and sequenced, and the locations of the mutations were mapped to gene *132* and *141* and the *141* to *142* intergenic region. The location of the AAA ATPase domain in gp141 (residues 186 to 323) predicted by pfam is shown. (D) DEM mapping in the Crossroads *141*-to-*142* intergenic region. The DNA sequence is shown for the beginning of the leftward-transcribed gene *141* and its amino acid sequence, together with the sequences immediately upstream. The ATG translation start codon for gene *141* is underlined, and the ribosome binding site (RBS) is indicated. Four DEM mutations are shown: a single base substitution at coordinate 74,303, 26-bp and 9-bp duplications, and a 99-bp deletion. (E) Crossroads *132* or *139* to *141* is required for targeting by Sbash *30*/*31*. Serial dilutions of a Crossroads mutant in which gene *132* is deleted (C’roadsΔ132) or genes 139 through 141 are deleted (C’roadsΔ*139–141*) together with Crossroads (C’roads), Sbash, and Pipsqueaks were plated onto lawns of M. smegmatis mc^2^155, a Sbash lysogen strain, and a pGG05 recombinant strain.

To understand how Crossroads is targeted for defense, we isolated a series of defense escape mutants (DEMs) that overcome Sbash gp30/gp31-mediated defense (Fig. 5A). A total of 24 mutants were isolated that escape defense by Sbash or by Sbash genes *30* and *31* (i.e., using plasmid pGG05), and the phage DNA was purified and sequenced (Table 1). Sequencing of the parental phage stock from which the lysates were prepared for mutant isolation revealed a 5,004-bp deletion (Δ38089–43093) that removed all or parts of genes *49* to *58*, which are evidently dispensable for lytic growth and are not involved in Sbash-targeting (Table 1); all of the mutants carried this deletion. The parental phage with the deletion was shown to be fully susceptible to Sbash defense. Relative to this parent phage, all 24 mutants contained single mutations that included small deletions or insertions and single nucleotide changes (Table 1) (Fig. 5B and C). Two pairs of mutants with the same mutations were isolated from the same lysates on the Sbash lysogen and the pGG05 recombinant (phgg344/phgg400 and phgg354/phgg408) and are presumably siblings (Table 1).

**TABLE 1:** Defense escape mutants of phage Crossroads

Mutant name	Defender	Length (bp)	Mutation	Gene (substitution)
Crossroads-wt		76,129		
Crossroads-par		71,124	Δ38089–43093	Δ*49*–*58*
phgg344	pGG05	71,124	G68548T	gp132 R185S
phgg348	pGG05	71,025	74303Δ99 bp	141–142 intergenic
phgg350	pGG05	71,124	A73608T	gp141 V214E
phgg354	pGG05	71,133	68435↑9 bp (dup)	gp132 222↑HDK
phgg356	pGG05	71,125	68575↑1 bp	gp132 175f/s
phgg359	pGG05	71,118	Δ73760–73765 (Δ6 bp)	gp141 ΔE161, ΔT162
phgg364	pGG05	71,124	Δ73760–73765 (Δ6 bp)	gp141 ΔE161, ΔT162
phgg398	Sbash	71,125	68395↑1 bp	gp132 236f/s
phgg399	Sbash	71,124	G68231T	gp141 A338D
phgg400	Sbash	71,124	G68548T	gp132 R185S
phgg403	Sbash	71,124	G73236T	gp141 A338D
phgg407	Sbash	71,124	G73275T	gp141 A325E
phgg408	Sbash	71,133	68435↑9 bp (dup)	gp132 222↑HDK
phgg409	Sbash	71,124	C73681A	gp141 V190F
phgg411	Sbash	71,124	G73317T	gp141 A311E
phgg413	Sbash	71,133	74335↑9 bp (dup)	141–142 intergenic
phgg417	Sbash	71,124	T74303C	141–142 intergenic
phgg419	Sbash	71,150	74312↑26 bp (dup)	141–142 intergenic
4_1A	Sbash	71,124	G73437A	gp141 S271F
3_4A	Sbash	71,118	Δ73760–73765 (Δ6 bp)	gp141 ΔE161, ΔT162
3_3A	Sbash	71,124	A73009G	gp141 *413Q
1_4A	Sbash	71,118	Δ73760–73765 (Δ6 bp)	gp141 ΔE161, ΔT162
1_3A	Sbash	71,118	Δ73760–73765 (Δ6 bp)	gp141 ΔE161, ΔT162
1_2A	Sbash	71,124	G73101T	gp141 A383E

Six of the DEM mutants mapped in Crossroads gene *132*, corresponding to four different mutations (Fig. 5C), two of which were single base insertions introducing frameshift mutations; one was a 9-bp duplication (dup) resulting in a three-residue insertion, and one introduced a single amino acid substitution (R185S) (Table 1) (Fig. 5C). The two frameshift mutations likely led to loss of function and the inability to be recognized by the Sbash gp30/31 system. Crossroads gene *132* was well conserved, and closely related homologues were present in 41 of the 44 cluster L phages. However, these (including Crossroads) contained a second, more distantly related homologue (e.g., Crossroads *140*; Fig. 5B), and Crossroads gp140 shared 32% amino acid identity with Crossroads gp132 (Fig. 5B). It is likely that neither is needed for lytic growth, as one cluster L phage (Bromden) has lost a large genome segment containing these genes. However, there are few bioinformatic clues as to their specific functions. Interestingly, several subcluster L2 phages had identical (e.g., Wilder, LilDestine, and Kahlid) or nearly identical (e.g., Faith1 and Gardann; one amino acid difference) homologues of Crossroads *132* but were not subject to Sbash defense (Fig. 4B and C).

**FIG 6:**
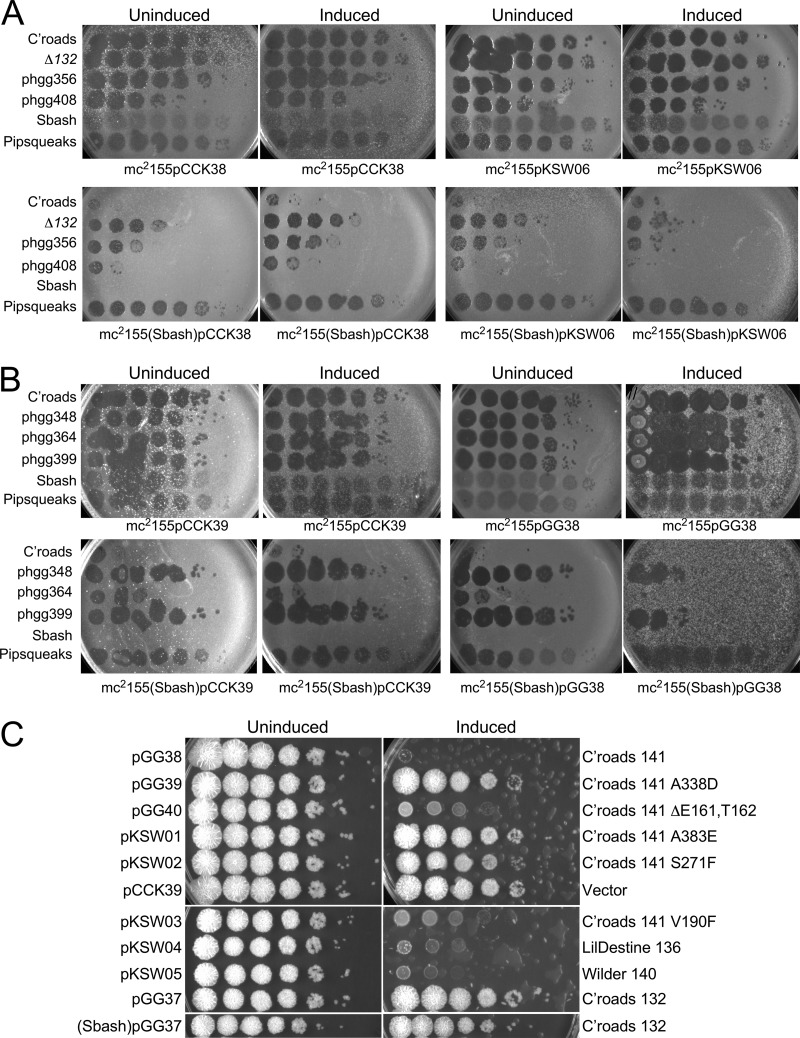
Expression of Crossroads gp132 and gp141. (A) Strains were constructed carrying extrachromosomal expression vector pCCK38 or plasmid pKSW06, in which Crossroads *132* expression can be induced by addition of ATc, in either M. smegmatis mc^2^155 (top row) or a Sbash lysogen (bottom row). Lawns were prepared on solid media with (Induced) or without (Uninduced) ATc, and 10-fold serial dilutions (from left to right) of phages were spotted. Phages tested were Crossroads, Crossroads Δ*132*, DEM mutants phgg356 and phgg408, Sbash, and Pipsqueaks. Expression of gp132 in mc^2^155(Sbash) results in an approximately 100-fold reduction in EOP of strain Δ*132* and the DEMs relative to the uninduced control. It is unclear why some of these phages have reduced plating efficiencies on M. smegmatis (Sbash) pCCK38 relative to M. smegmatis mc^2^155pCCK38. (B) Strains containing integration-proficient vector pCCK39 or plasmid pGG38 in which Crossroads *141* can be induced with ATc were plated as described in the panel A legend, except the ATc concentration was reduced to 5 ng/ml and the plates were incubated for 3 days (all others were incubated for 1.5 days). Phages Crossroads, phgg348, phgg364, phgg399, Sbash, and Pipsqueaks were spotted as described in the panel A legend. Expression of gene *141* substantially reduced the plating efficiencies of DEM mutants in a Sbash lysogen. (C) Liquid cultures of strains containing the plasmids indicated on the left were grown to an OD_600_ of 0.5, serially diluted 10-fold, and spotted onto solid media with (Induced) or without (Uninduced) ATc. The genes/mutants in each plasmid are noted at the right. Plasmids pGG37 and pKSW06 are integrating and extrachromosomal plasmids, respectively, containing Crossroads *132*, and neither displayed toxicity in either a Sbash lysogen or a nonlysogen.

To examine the role of Crossroads *132*, we used BRED engineering to construct a Crossroads deletion mutant (Crossroads Δ*132*). The Δ*132* mutant grew well and formed plaques that were similar in size to those seen with wild-type Crossroads, demonstrating that *132* is not required for lytic growth (Fig. 5E). The Δ*132* mutant plated with an EOP value of 1 on both the Sbash lysogen and a recombinant strain carrying pGG05, showing that *132* was required for Sbash *30*/*31* targeting (Fig. 5E). Expression of Crossroads *132* from a recombinant strain carrying plasmid pKSW06 (see Table S2 in the supplemental material) at least partially restored Sbash-mediated defense of the Crossroads Δ*132* mutant and DEMs phgg356 and phgg408 (Fig. 6A), confirming that the escape phenotype resulted from loss of gp132 function.

Fourteen of the mutants mapped in Crossroads gene *141*, whose specific function is unknown but is related to AAA ATPases (Walker A box residues at 191 to 198; Fig. 5B). The mutants correspond to nine different mutations, one of which is a 6-bp deletion removing codons 161 and 162 and was isolated independently five times (Table 1) (Fig. 5B). Oddly, one mutation is within the termination codon and a single nucleotide change introduces a glutamine codon, enabling readthrough and inclusion of 28 additional C-terminal amino acids. Seven mutations introduced single amino acid substitutions, primarily within the C-terminal half of the predicted gp141 protein (Fig. 5B). The remaining four mutants mapped to the 370-bp *141*-to-*142* intergenic region (Fig. 5D) and included a 99-bp deletion, duplications of 9 bp or 26 bp, and a single nucleotide substitution at coordinate 74303 (Fig. 5B). All of these changes were upstream of the *141* start codon (comparative analysis and coding potential strongly support the use of this start codon), and these mutations presumably influence *141* expression rather than gp141 activity *per se*.

Closely related homologues of Crossroads *141* were present in 37 of the 44 sequenced cluster L phages, but their absence from seven of the phages, including the subcluster L2 phage BigCheese (Fig. 4A and B), indicated that it is not required for lytic growth. Interestingly, none of the *141* DEM mutants have large deletions or frameshift mutations within the open reading frame, and none of the single amino acid substitutions are in the Walker A motif or other known catalytic residues (Fig. 5C). Six of the substitutions were in residues that are highly conserved among the mycobacteriophage homologues, four of which are conserved alanines (A311E, A325E, A338D, and A383E). Attempts to knock out *141* from Crossroads were unsuccessful, suggesting that although it is not required for lytic growth, eliminating it resulted in a nonviable phenotype. Thus, the *141* DEM phenotypes likely resulted from reductions in expression or activity rather than from complete loss of function. Phage BigCheese, which lacks *141*, has also lost genes *139* and *140* (Fig. 4B), suggesting that loss of one or both of these may rescue the nonviable phenotype of a *141* null mutant. We thus attempted to knock out *140* and *141* together, as well as *139* through *141*. We were unable to recover a knockout of *140* and *141*, but a knockout of *139* to *141* (mimicking the BigCheese genome arrangement; Fig. 4B) was readily obtained. Therefore, viable inactivation of *141* requires loss of *139* and possibly of both *139* and *140*. We then tested the ability of the Crossroads Δ*139–141* mutant to escape Sbash *30*-to-*31*-mediated defense. The Crossroads Δ*139–141* mutant grew to a high titer, formed plaques that were similar in size to those seen with wild-type Crossroads, and plated with an EOP value of 1 on both a Sbash lysogen and a pGG05 recombinant strain (Fig. 5E). Taken together, these observations are consistent with the interpretation that escape from Sbash can be achieved by mutational reduction of gp141 activity or by altering its expression by mutation in the *141*-to-*142* intergenic region. Escape from defense can also occur by loss of gp141 but requires accompanying loss of gp139 and gp140.

Attempts at complementation of the *141* DEM defect are complicated by the toxicity of Crossroads *141* when expressed in M. smegmatis, even in the absence of Sbash (Fig. 6B and C). However, by using very small amounts (5 ng/ml) of anhydrotetracycline (ATc) and extended incubation times (Fig. 6B), we could show that *141* expression (from plasmid pGG38, which contains an ATc-inducible *141* gene) in a Sbash lysogen substantially restored the reduced EOP of DEMs phgg348, phgg364, and phgg399 (Fig. 6B), consistent with the interpretation that reduced expression or activity of Crossroads gp141 is directly responsible for the escape phenotype.

### Only some DEM mutants of Crossroads 141 are toxic in M. smegmatis.

Expression of Crossroads gp132 in M. smegmatis was well tolerated, using induction of expression with 100 ng/ml ATc (Fig. 6C). Furthermore, expression of gp132 in a Sbash lysogen was also well tolerated (Fig. 6C). The Sbash *30*/*31* defense thus seems distinct from those abortive infection (*abi*) mechanisms that involve toxin-antitoxin-mediated cell death (24, 25), although Crossroads gp132 alone could be insufficient to activate growth inhibition. Expression of Crossroads gp141 in nonlysogenic M. smegmatis was highly toxic, and growth was strongly inhibited (Fig. 6C), even in the absence of the Sbash prophage. Interestingly, in testing five different DEM mutants with mutations within *141*, two (Δ161 and Δ162; V190F) exhibited substantial toxicity—albeit milder than that seen with wild-type *141*—and three (A338D, A383E, and S271F) showed little or no toxicity at all. We also tested expression of the Crossroads *141* homologues in phages Wilder (*140*) and LilDestine (*136*), both of which were found to be toxic when expressed (Fig. 6C). Taken together, these observations suggest that the toxicity of Crossroads gp141 is unrelated to its role in targeting Crossroads for defense by the Sbash system.

### RNAseq profiles of Crossroads and DEM mutants.

**FIG 7:**
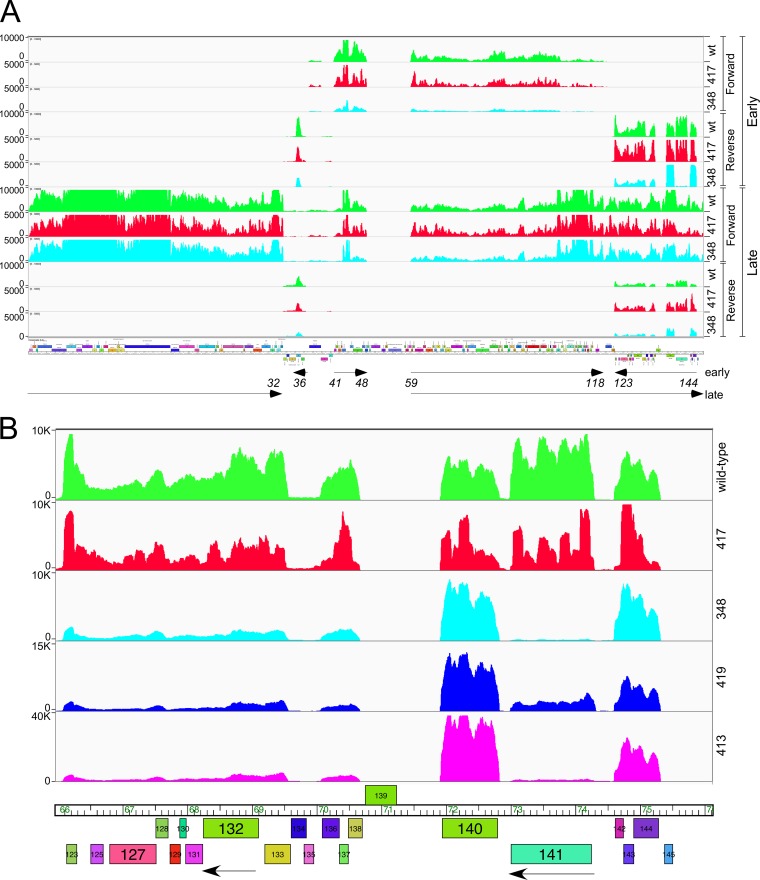
RNAseq analysis of Crossroads and DEM mutants. (A) RNA was isolated at early (30 min) and late (150 min) time points after infection of M. smegmatis with wild-type Crossroads (green) or DEMs phgg348 (aqua) and phgg417 (red); reads mapping to forward and reverse strands are shown as indicated. Arrows at the bottom indicate the early and late transcribed regions, with gene numbers denoting the positions. (B) Expanded view of RNAseq reads mapping to the reverse strands at the right end of the genome. DEM mutants phgg419 (blue) and phgg413 (magenta) are shown in addition to those shown in panel A and are similarly colored. Arrows indicate leftward transcription of the two genes, *132* and *141*, in which DEM mutants mapped. Reads are shown for the reverse strand only.

To examine the impact of the DEM mutations mapping upstream of *141* on gene expression, we analyzed the RNAseq profiles of wild-type Crossroads and DEMs phgg348, phgg413, phgg417 and phgg419 (Table 1) (Fig. 7A), at 30 min and 150 min after infection, which we refer to here as “early” and “late” time points, respectively. In wild-type Crossroads, many of the leftward-transcribed genes at the right end of the genome (genes *122* to *144*) were expressed early, together with gene *36* encoding a Lsr2-like protein (Fig. 7A). Rightward early transcription starts just upstream of gene *41* and transcribes genes *41* to *48* and also occurs at a location near gene *58* to transcribe genes *59* to *118*. At the late time point, early transcripts were still detected, but rightward transcription proceeded from upstream of gene *59* all the way through to the right genome end; the virion structure and assembly genes, especially genes *6* to *16*, were highly expressed also (Fig. 7A). It is unusual that transcripts were present for both strands of genes *122* to *144* at the late time point (Fig. 7A).

Transcription of gene *141* initiated in the *141*-to-*142* intergenic region (Fig. 7B), and the deletion mutant (phgg348) and both insertion mutants (phgg413 and phgg419) (Table 1) (Fig. 5C) showed strongly reduced transcription of gene *141* (Fig. 7B), particularly in mutants phgg348 and phgg413, consistent with mutational interruption of the promoter (Fig. 7B); there was also an apparent modest reduction in expression of genes *125* to *138* (Fig. 7). Transcription of *141* was not substantially reduced in the phgg417 point mutant (Fig. 7B), and it is plausible that the DEM phenotype resulted from inefficient translation of gp141. However, the mutation was 55 bp upstream of the predicted translation start site (Fig. 5C) and thus beyond the normal location of a ribosome binding site (RBS).

### Mechanism of Sbash *30*-to-*31*-mediated defense against Crossroads.

**FIG 8:**
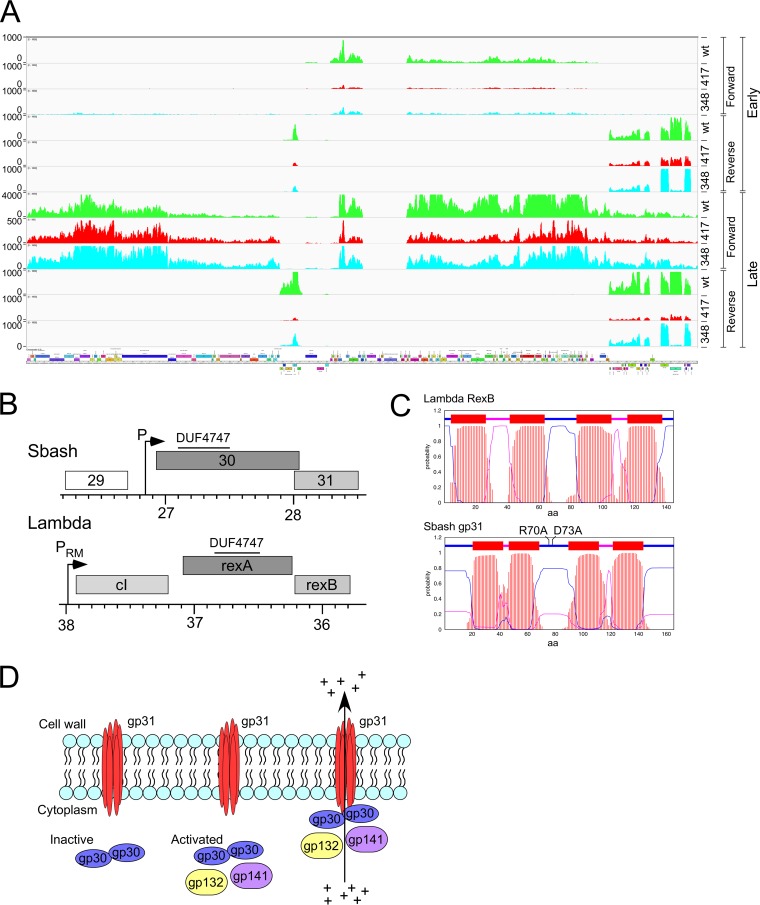
Mechanism of Sbash 30-to-31-mediated defense against Crossroads. (A) RNAseq analysis of Crossroads and DEM mutant infection of M. smegmatis mc^2^155pGG05. RNA was isolated at early (30 min) and late (150 min) time points after infection of M. smegmatis mc^2^155pGG05 with wild-type Crossroads (green) or DEMs phgg417 (red) and phgg348 (aqua); reads mapping to forward and reverse strands are shown as indicated. Scales were adjusted to reflect approximately similar levels of early gene expression. (B) Comparison of Sbash and lambda genomes. Segments of the two phage genomes were aligned to show similarities between Sbash defense genes *30* and *31* and lambda exclusion genes *rexA* and *rexB*. The positions of promoters expressing Sbash *30*/*31* and lambda *cI*/*rexAB* are shown, and the locations of a DUF4747 conserved domain in Sbash gp30 and lambda RexA are also indicated. The function of Sbash gp29 is not known, but it likely a virion tail gene; the lambda gene orientation has been reversed for illustration purposes. (C) TMHMM prediction of membrane topology of Sbash gp31 and RexB. Probabilities of amino acids being located within the membrane and cytoplasm and externally are shown in red, purple, and blue, respectively. The positions of two separate amino acid substitutions in Sbash gp31 that inactivate defense are shown (see Fig. S2B). (D) A model for Sbash 30/31-mediated defense. Sbash gp31 is proposed to be membrane located but inactive as an ion channel until infection with phage Crossroads. During early lytic growth of phage Crossroads, gp132 and gp141 act either directly or indirectly through Sbash gp30 to activate the gp31 ion channel, leading to loss of membrane potential and of intracellular ATP, interruption of macromolecular synthesis, and loss of cell viability. It is not known if Sbash gp30 interacts directly with gp31 in either the inactive or the activated state, but substitutions in the central cytoplasmic loop of Sbash gp31 are inactive for defense, consistent with an interaction between Sbash gp30 and gp31.

To explore how Sbash *30*/*31* defends against Crossroads infection, we first used RNAseq to determine if Sbash *30* to *31* inhibits an early stage of infection or after DNA injection occurs and gene expression begins. When Crossroads infected a strain expressing Sbash genes *30* and *31* (Fig. 8A), the early expression pattern was similar to that seen with Crossroads transcription following infection of wild-type M. smegmatis (Fig. 7A). Thus, there was no indication of an early block to adsorption, DNA injection, or early transcription (Fig. 8A). However, at the late time point, there was a notable reduction in late gene expression, reflected in the number of RNAseq reads mapping to the virion structure and assembly genes (*1* to *32*) relative to transcripts present at that time but expressed early (e.g., genes *59* to *118*). In infection of wild-type cells, the ratio of Crossroads late transcripts to early transcripts at the late time point was 3.88 (Fig. 7A), but the late transcript/early transcript ratio in the presence of Sbash *30*/*31* at the late time point was only 0.61 (Fig. 8A). DEM mutants phgg348 and phgg417 had late transcript/early transcript ratios similar to the those seen with the wild type at late infection of wild-type M. smegmatis (4.82 and 3.02, respectively), but in a Sbash *30*/*31* host they at least partially recovered (late transcript/early transcript ratios of 1.75 and 1.71, respectively) from the defect seen in wild-type Crossroads (Fig. 8A).

10.1128/mBio.00196-19.2 FIG S2 ClustalX alignment of Sbash gp31 and four homologues. (A) Sbash gp31 was aligned with *Gordonia* phage CarolAnn gp44 (YP_009291998.1) and hypothetical proteins of *Rhodococcus* sp. 29MFTsu3.1 (WP_019666843.1), Mycobacterium fortuitum (WP_065068565.1), and Mycobacterium setense (WP_074873798.1) using ClustalX. Genes corresponding to the latter three are all adjacent to homologues of Sbash gp30 genes and are likely to be prophage encoded. The four transmembrane domains (TM1 to TM4) predicted by TMHMM are shown as boxes on the Sbash gp31 sequence. Although there are few amino acid residues conserved in all five proteins (indicated by asterisks), most of them are in the putative cytoplasmic domain between TM2 and TM3. Vertical arrows indicate residues at which mutant substitutions were constructed. (B) Lysates of phages Giles, Crossroads, Sbash, and Pipsqueaks were serially diluted 10-fold and spotted onto lawns of M. smegmatis mc2155, Sbash lysogen [mc2155(Sbash)], a strain carrying pGG05, and strains carrying plasmid pGG12 or plasmid pGG13, each of which is a derivative of plasmid pGG05, with mutations conferring either R70A or D73A in Sbash gp31, respectively. Download FIG S2, PDF file, 0.4 MB. Copyright © 2019 Gentile et al. 2019 Gentile et al. This content is distributed under the terms of the Creative Commons Attribution 4.0 International license.

Sbash genes *30* and *31* share features with the genes encoding the RexAB system of phage lambda (Fig. 8B); Sbash gp31 and RexB both have four predicted transmembrane domains (Fig. 8C), and Sbash gp30 and lambda RexA both contain a DUF4747 motif (Fig. 8B). *RexAB* excludes infection of T4*rII* mutants and is thought to do so by RexA-mediated activation of the ion channel potential of RexB (26, 27). The Sbash system may act similarly, such that when activated, Sbash gp31 mediates membrane depolarization, therefore lowering intracellular ATP concentrations and causing interruption of macromolecular synthesis and abortion of phage growth (Fig. 8D). The RNAseq profiles are consistent with this model, and we similarly observed a reduction in phage DNA amplification. In Crossroads infection of M. smegmatis, ∼90% of DNA sequencing reads mapped to the Crossroads genome, whereas in infection of a Sbash *30*-to-*31*-expressing strain, fewer than 5% of reads mapped to Crossroads. The interruption of both phage DNA replication and late transcription is consistent with a reduction in intracellular ATP concentration.

The model proposes that the membrane-located Sbash gp31 acts as an ion channel when stimulated by Sbash gp30, which in turn is activated by Crossroads infection. The predicted gp31 topology suggests there are N- and C-terminal segments that are cytoplasmically located together with a region between the second and third membrane-spanning domains (Fig. 8C; see also Fig. S2A). Database searches identified four Sbash gp31 homologues and showed that although few residues are highly conserved, there are eight invariant positions in the putative central cytoplasmic region (Fig. S2A). We constructed two separate mutants (R70A and D73A) with substitutions in this region (Fig. 8C); both were defective in defense (Fig. S2B). This suggests that this is a functionally important part of gp31, and we propose that it is involved in Sbash gp30 interactions.

### The *Gordonia* phage CarolAnn gp44/gp43 defense system also targets Crossroads.

**FIG 9:**
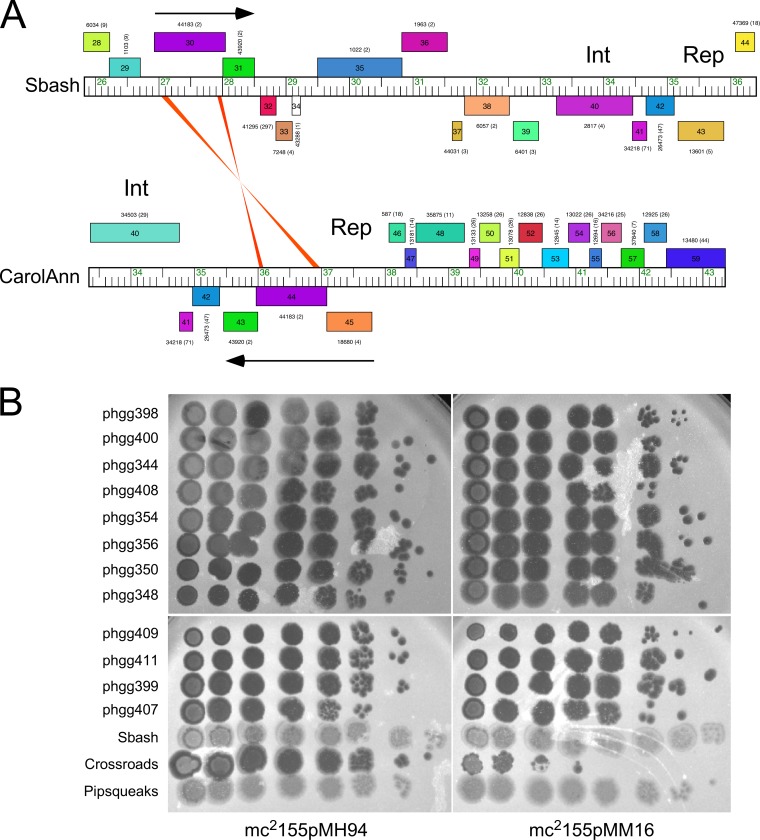
CarolAnn genes *43* and *44* confer defense against Crossroads. (A) Genome comparison of mycobacteriophage Sbash and Gordonia phage CarolAnn. Shading between the genomes reflects nucleotide sequence similarities, although there are only two short regions of weak similarity at the extremities of Sbash *30* and CarolAnn *43*. Sbash gp30/CarolAnn gp44 and Sbash gp31/CarolAnn gp43 homologous pairs share 50% and 43% amino acid identity, respectively. Sbash *30* and *31* are transcribed rightward and are displaced from the repressor gene (gene *43*), whereas CarolAnn *43* and *44* are coexpressed with the repressor (gene *45*). (B) Serial dilutions of Sbash, Crossroads, Pipsqueaks, and 12 Crossroads DEM mutants (labeled phggXXX) were plated onto lawns of M. smegmatis mc^2^155pMH94 (vector) and M. smegmatis mc^2^155pMM16, which carries CarolAnn genes *43* and *44*.

There are no homologues of Sbash genes *30* and *31* elsewhere in the extant collection of mycobacteriophage genomes, but there is a pair of related genes in *Gordonia* phage CarolAnn (see accompanying paper [33]) (Fig. 9A). CarolAnn gp44 and gp43 share 50% and 42% amino acid identity with Sbash gp30 and gp31, respectively, and confer defense against infection of several heterotypic *Gordonia* phages, including Kita and Nymphadora. However, these Gordonia phages are not related to Crossroads and do not have homologues of Crossroads *132* and *141.* Instead, Kita and its relatives are targeted for defense through Kita gene *53* and its homologues, which are of unknown function, and are not present in Crossroads. To examine the functional relationship between the two defense systems, plasmid pMM16 expressing CarolAnn *43* and *44* was transformed into M. smegmatis and tested for Crossroads infection (Fig. 9B). Somewhat surprisingly, CarolAnn *43*/*44* conferred defense not only against Crossroads but also against the Crossroads DEMs—including those in gene *132*, in gene *141*, or in the *141*-to-*142* intergenic region efficiently escaping the defense system (Fig. 9B). We have also shown that the Sbash genes *30* and *31* confer defense against *Gordonia* phage Kita when expressed in Gordonia terrae (see accompanying manuscript [33]). Sbash gp30/gp31 and the CarolAnn gp43/gp44 systems thus act similarly in M. smegmatis and G. terrae, even though they recognize different and unrelated target phages.

## DISCUSSION

We have described here a new viral defense system encoded in mycobacteriophage Sbash, which is expressed lysogenically and defends against phage Crossroads with remarkable specificity. Viral defense plays a central role in bacterial survival in most environments, and Sbash colludes with its M. smegmatis bacterial host to mutually benefit the phage and the host. There is at least one other Sbash defense system responsible for defense against phage Giles, and the other subcluster I2 phage, Che9c, codes for at least one additional defense system. It seems probable that such prophage-mediated defense systems are widespread across the bacterial landscape of diversity, and the variety of systems suggests that there is a vast reservoir of such defenses remaining to be discovered. These likely dwarf the number of bacterially encoded systems reported to date (7).

We propose a mechanism of Sbash *30*/*31* defense reflecting the proposed mechanism for lambda RexAB exclusion of T4*rII*; Sbash gp30 is activated by Crossroads infection, leading to stimulation of the ion channel potential of gp31, membrane depolarization, and loss of intracellular ATP (Fig. 8D). As a consequence, lytic growth is inhibited and the infection aborts. Consistent with this is the observation that early gene expression of Crossroads proceeds in the presence of Sbash *30*/*31*, indicating that there is not a block to DNA injection or initiation of the transcriptional program (Fig. 7). Reduced levels of transcription and DNA replication occur at later time points, consistent with an overall inhibition of macromolecular synthesis (Fig. 8A). Whereas the lambda RexAB system is thought to be activated by binding of DNA, this seems unlikely for Sbash *30*/*31*, as Crossroads gp132 and gp141 are both required for targeting of Crossroads, and neither is essential for lytic growth. It is plausible that gp132 and gp141 act to directly interact with Sbash gp30 to stimulate gp31 ion channel activity (Fig. 8D).

It is striking that the homologous defense system from *Gordonia* phage CarolAnn confers defense against Crossroads in M. smegmatis and that, reciprocally, the mycobacterial Sbash system defends against phage Kita (and its relatives) in *Gordonia* (see accompanying paper [33]). In each instance, defense escape mutants isolated in the cognate system also escape the noncognate system. However, the targeted phages (Crossroads, Kita) are unrelated, and the escape mutants map in entirely different genes. This emphasizes the complexity of these defense systems and how they can respond to different types of attacking phages. It is also noteworthy that Kita gp53 expression leads to growth inhibition in the presence of CarolAnn *43*/*44*, consistent with an abortive infection system as described above (see accompanying paper [33]). Further studies are required to determine the mechanism of activation in these different systems and whether Crossroads and Kita activate via similar mechanisms notwithstanding their sequence differences.

The Sbash prophage targets Crossroads with remarkable specificity, and none of the other closely related subcluster L2 phages are similarly defended against (Fig. 4B). It is striking that phages Wilder, LilDestine, and Crossroads have identical copies of Crossroads *132* and that the *141* homologues differ by five or fewer substitutions. One plausible explanation is that these differences reflect the evolutionary dynamics differing between the prophage-mediated defense systems and the infecting phages, with selection of defense and counterdefense systems, respectively. Thus, it is possible that Crossroads is the outlier and that—unlike the other subcluster L2 phages—it has not yet acquired a mechanism to escape the Sbash system, even though mutants that do so can readily be isolated.

Finally, we note that although phages Sbash and CarolAnn are the only two phages in the collection of 2,800 sequenced actinobacteriophages that encode these defense systems, there are several sequenced isolates of Mycobacterium fortuitum, Mycobacterium immunogenum, and *Rhodococcus* sp. that have homologues of Sbash gp30 and that also encode a small membrane protein immediately downstream; most are likely encoded by prophages (see Fig. S2 in the supplemental material). This suggests there may be a much larger family of defense systems that show organization and operation similar to those of lambda *rexAB* and whose members have yet to be fully explored.

## MATERIALS AND METHODS

### Strains and plasmids.

M. smegmatis mc^2^155 (29) was used for phage growth and analyses as described previously (4). Phage lysates typically had titers greater than 10^9^ PFU ml^−1^ and were used for plaque assays and DNA extraction. Lysogens were isolated by spotting 10 µl of high-titer lysate onto a lawn of M. smegmatis mc^2^155, incubating at 37°C for 18 to 24 h, and streaking for isolated colonies from the infected area. Individual colonies were propagated and tested for phage release and immunity to superinfection.

Primers and plasmids used in this study are shown in Tables S1 and S2 in the supplemental material, respectively. Plasmid vectors pJV39 and pGH1000 are hygromycin (Hyg)-resistant, integrated vectors containing the integration cassette of mycobacteriophage L5 and Giles, respectively (30, 31). Cloning of genes into vectors pJV39 and pGH1000 was done using Gibson assembly (New England Biolabs, Inc.) with PCR-amplified substrates inserted at the vector HindIII restriction site. Plasmid vector pCCK39 is a streptomycin (STR)-resistant integrated vector containing the integration cassette of mycobacteriophage L5 (32). The pCCK39 backbone is a Tet-ON plasmid (34) containing an inducible promoter; derivatives of pCCK39 were constructed to include 20 bp upstream of the gene(s) of interest to include the native RBS. Cloning of genes into pCCK39 was done using Gibson assembly (New England Biolabs, Inc.) with PCR-amplified substrates inserted at the vector PmlI restriction site. Gene expression of pCCK39 derivatives was induced by addition of 100 ng ml^−1^ of anhydrotetracycline (ATc) to solid media, unless otherwise indicated. Plasmid vector pCCK38 is a Hyg-resistant extrachromosomal vector that contains *oriM*, and plasmid pKSW06 was constructed in a manner akin to that used to construct the pCCK39 derivatives described above. All phage-derived DNA fragments to be cloned were amplified from phage Sbash or phage Crossroads genomic DNA using oligonucleotide primers as shown in Table S1. Plasmid DNAs were extracted from Escherichia coli and transformed into electrocompetent M. smegmatis mc^2^155 cells. All constructed plasmids were verified by DNA sequencing. Site-directed mutants were constructed using a Q5 site-directed mutagenesis kit (New England Biolabs, Inc.) to construct plasmid pGG24, in which gene *30* is deleted from plasmid pGG05.

10.1128/mBio.00196-19.3 TABLE S1 Oligonucleotide primers used in this study. Download Table S1, PDF file, 0.02 MB. Copyright © 2019 Gentile et al. 2019 Gentile et al. This content is distributed under the terms of the Creative Commons Attribution 4.0 International license.

10.1128/mBio.00196-19.4 TABLE S2 Plasmids constructed in this study. Download Table S2, PDF file, 0.03 MB. Copyright © 2019 Gentile et al. 2019 Gentile et al. This content is distributed under the terms of the Creative Commons Attribution 4.0 International license.

### Phage growth and analyses.

High-titer phage lysates were prepared by diluting a lysate 10-fold into phage buffer with 0.1 M CaCl_2_ and plating 10 µl of the 10^−2^ dilution with 250 µl of mc^2^155 and 3 ml of liquid Middlebrook 7H9 medium. All plates were incubated at 37°C for 18 to 24 h. Webbed plates were flooded with 4 ml of phage buffer and incubated at room temperature for at least 4 h. Lysates were collected, filtered through a 0.22 μm-pore-size filter, and then stored at 4°C. The phage genome sequences have been reported previously or are available in GenBank. A Phamerator database (Actinobacteriophage_Draft, August 2018) was used for comparative genomic analyses (35). Phage genomic DNA was extracted using either a Wizard DNA kit (Promega) or phenol-chloroform as described previously (36) and was completely sequenced using Illumina MiSeq 150-base single-end runs and assembled as described previously (11).

### Isolation and characterization of defense escape mutants (DEMs).

Defense escape mutants (DEMs) were identified by plating dilutions of independently prepared lysates onto lawns of M. smegmatis mc^2^155 lysogenic strains or onto lawns of a strain carrying plasmid pGG05 (Table S2). Individual plaques of defense escape mutants were recovered from independently prepared lysates, purified, and amplified. Phage DNAs were isolated and sequenced.

### Construction of phage mutants.

Phage mutants were constructed using Bacteriophage Recombineering of Electroporated DNA (BRED) as previously described (37). An approximately 500-bp gBlock DNA fragment (Table S3; Integrated DNA Technologies, Inc.) was synthesized and PCR amplified using ∼20-bp flanking primers and Q5 high-fidelity DNA polymerase (New England BioLabs, Inc.). This DNA substrate was coelectroporated with phage genomic DNA (150 to 300 ng) into electrocompetent recombineering cells (M. smegmatis mc^2^155pJV53). Following recovery for 1 h with shaking at 37°C in 7H9 medium–10% ADC (0.5% Bovine Serum Albumin, 0.2% Dextrose, 0.85% NaCl)–1 mM CaCl_2_, the cells were plated with 7H9 top agar and wild-type M. smegmatis mc^2^155 cells. Primary plaque picks were screened by PCR to identify those containing the mutant allele, and mixed plaques were picked, diluted, and replated. Secondary plaques were picked and screened by PCR, followed by amplification.

10.1128/mBio.00196-19.5 TABLE S3 DNA substrates and primers using for phage engineering. Download Table S3, PDF file, 0.03 MB. Copyright © 2019 Gentile et al. 2019 Gentile et al. This content is distributed under the terms of the Creative Commons Attribution 4.0 International license.

### RNAseq analysis.

For RNAseq analysis, RNA was isolated from M. smegmatis lysogens in late logarithmic growth or from phage-infected cells (multiplicity of infection of 3) at 30 or 150 min after infection. Following DNA removal and rRNA depletion performed using a Turbo DNA-free kit (Ambion) and a Ribo-Zero kit (Illumina), respectively, a TruSeq stranded RNAseq kit (Illumina) was to used to prepare libraries. These were evaluated using a BioAnalyzer and run on an Illumina MiSeq system. The fastq reads were analyzed as described previously (38). The Integrative Genomics Viewer (39) was used to visualize and present the RNAseq coverage. RNAseq data sets, with additional method details, are deposited in the Gene Expression Omnibus (GEO) with accession number GSE121829. The average read length was 140 bases. To determine the ratios of late transcripts to early transcripts at the late time point, the number of reads mapping to late genes (genes *1* to *32* and *96* to *113*, and genes on the strand opposite that harboring genes *125* to *138* and *140* to *145*) was divided by the number of reads mapping to early genes (genes *36* to *37*, *41* to *48*, and *59* to *118*). The ratios were calculated within each sample; thus, the variation of number of reads between each sample (likely due to differences in infection efficiency) did not factor into the calculation.

### Toxicity analysis.

Plasmids were constructed as outlined above, and inducible clones were transformed into strains of interest by electroporation. Transformed cultures were grown to saturation in the presence of STR and were diluted and grown with STR to an optical density at 600 nm (OD_600_) of 0.5. Cultures were then serially diluted 10-fold, and the neat to 10^−7^ dilutions were spotted onto 7H11-STR agar plates with (Induced) and without (Uninduced) 100 ng/ml ATc. Plates were incubated for 3 days at 37°C and imaged.

### Defense complementation analysis.

M. smegmatis mc^2^155 and mc^2^155(Sbash) strains containing pKSW06 or pGG38 or empty vector were grown to saturation in the presence of selection, and lawns were poured on 7H10-Hyg or 7H11-STR plates with (Induced) and without (Uninduced) ATc; 100 ng/ml ATc was used for the strains containing pKSW06 and relevant controls, while 5 ng/ml ATc was used for the strains containing pGG38 and relevant controls. Phage lysates were serially diluted 10-fold and spotted onto lawns, and plates were incubated at 37°C for 1.5 days, except for the strains containing pGG38, which were incubated for 4 days, and then imaged.

### Data availability.

The RNAseq data sets, with additional method details, have been deposited in the Gene Expression Omnibus (GEO) with accession number GSE121829. All phage genome sequences are available at https://phagesdb.org.
