# Supplementary figures and images for: More Evidence of Collusion: a New Prophage-Mediated Viral Defense System Encoded by Mycobacteriophage Sbash
Source: mBio. 2019 Mar 19;10(2):e00196-19. doi: 10.1128/mBio.00196-19 (PMC6426596; doi:10.1128/mBio.00196-19)

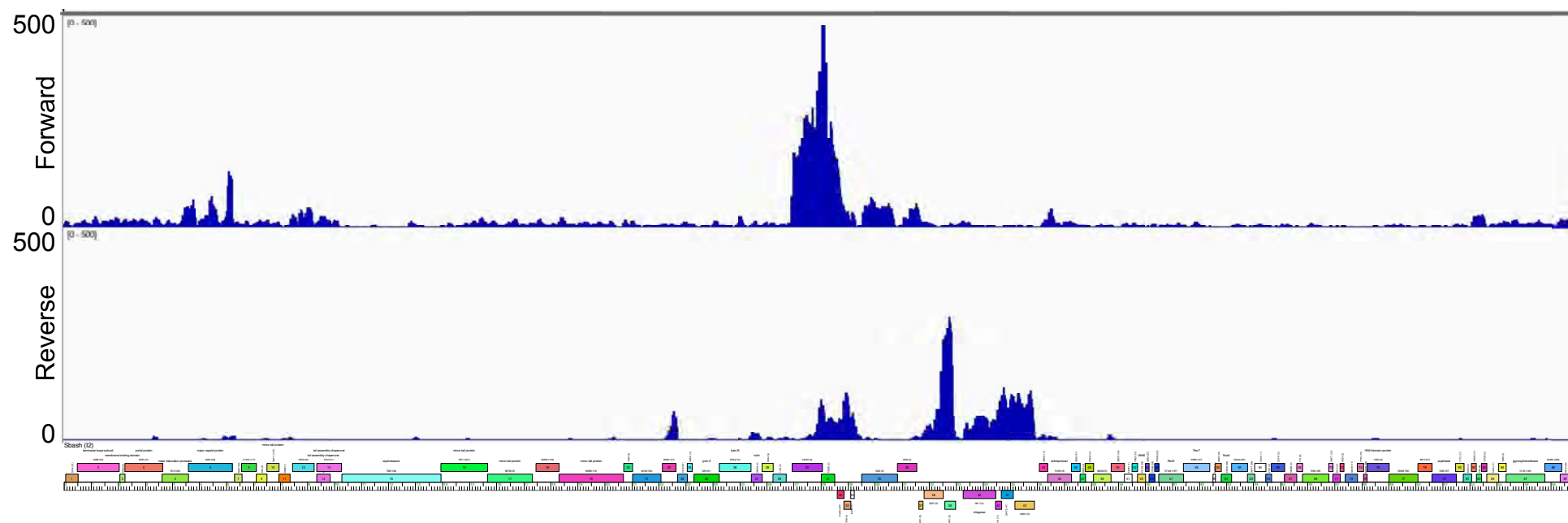

Figure S1

Supplement: FIG S1 [file mBio.00196-19-sf001.pdf]
