# Supplementary material for: More Evidence of Collusion: a New Prophage-Mediated Viral Defense System Encoded by Mycobacteriophage Sbash
Source: mBio. 2019 Mar 19;10(2):e00196-19. doi: 10.1128/mBio.00196-19 (PMC6426596; doi:10.1128/mBio.00196-19)
Supplement: FIG S2 [file mBio.00196-19-sf002.pdf]

A

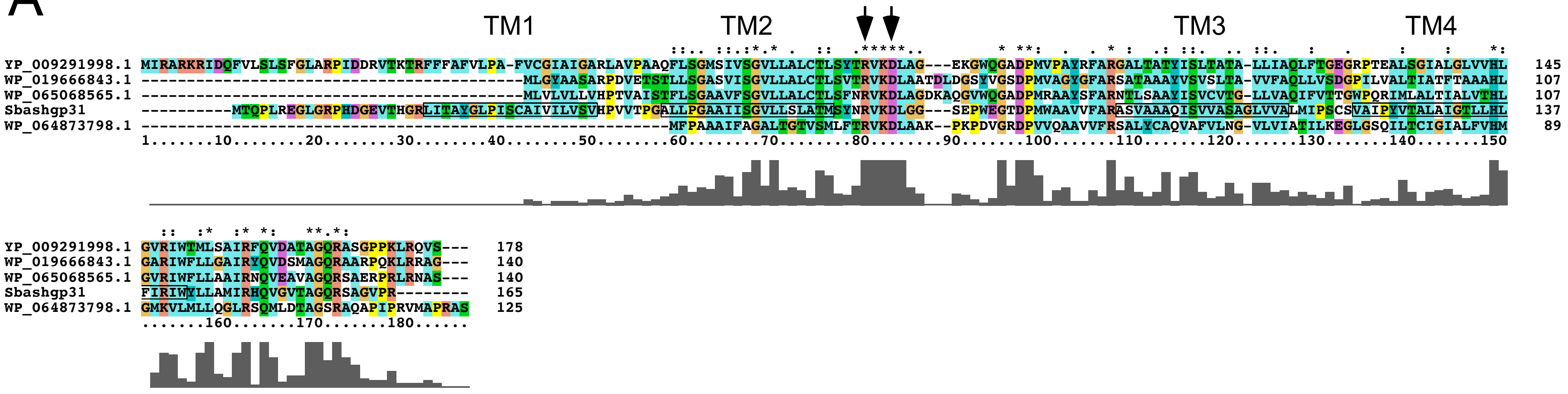

TM1
TM2
TM3
TM4
YP_009291998.1
WP_019666843.1
WP_065068565.1
Sbashgp31
WP_064873798.1

B

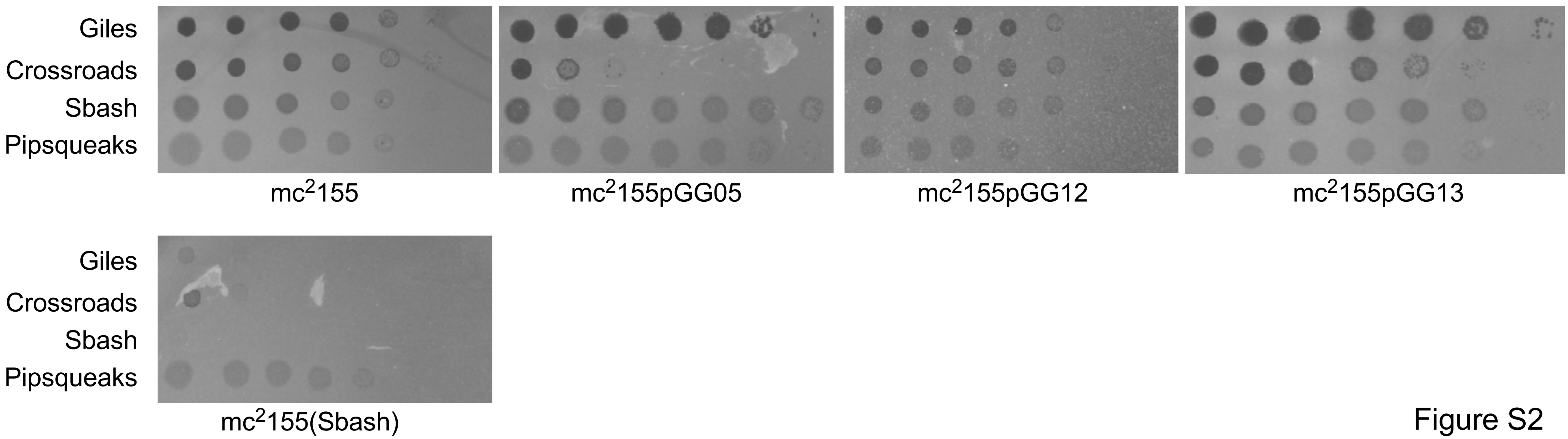

Giles
Crossroads
Sbash
Pipsqueaks
mc^{2}155
mc^{2}155pGG05
mc^{2}155pGG12
mc^{2}155pGG13
Giles
Crossroads
Sbash
Pipsqueaks
mc^{2}155(Sbash)

Figure S2
