# Supplementary material for: More Evidence of Collusion: a New Prophage-Mediated Viral Defense System Encoded by Mycobacteriophage Sbash
Source: mBio. 2019 Mar 19;10(2):e00196-19. doi: 10.1128/mBio.00196-19 (PMC6426596; doi:10.1128/mBio.00196-19)
Supplement: TABLE S1 [file mBio.00196-19-st001.pdf]

Table S1. Oligonucleotide primers used in this study

Primer	Sequence
pGG01 FWD	ATACGACTCACTATAGGGAAATCGACAACCGCCCCCGAC
pGG01 REV	AGTCGACCTGCAGGCATGCACGGCGGCCATCGACCCCT
pGG02 FWD	ATACGACTCACTATAGGGAAAACTAAGGGTGTAGGTCAAC
pGG02 REV	AGTCGACCTGCAGGCATGCAGTGCAGGATTCGAACCTG
pGG03 FWD	ATACGACTCACTATAGGGAAACGCAATGACACGCATATAAATACTGGTGGG
pGG03 REV	AGTCGACCTGCAGGCATGCACCCGCCTCCCCCAAAGCC
pGG04 FWD	ATACGACTCACTATAGGGAATTCCTCCTTTCCGCCTGC
pGG04 REV	AGTCGACCTGCAGGCATGCACACACAGGAGATTTGAGTACGAC
pGG05 FWD	ATACGACTCACTATAGGGAACCGGCGAACCCATCGACAAC
pGG05 REV	AGTCGACCTGCAGGCATGCAGGCATTCGGCGGCCATCG
pGG06 FWD	ATACGACTCACTATAGGGAACGCGCATGCCTTTGCCCT
pGG06 REV	AGTCGACCTGCAGGCATGCAAACCAAAACAGGCCCCCTC
pGG07 FWD	ATACGACTCACTATAGGGAAGCCCGATGAAGCTAGATG
pGG07 REV	AGTCGACCTGCAGGCATGCAATCCCCGAAGATACACCG
pGG08 FWD	ATACGACTCACTATAGGGAATCAGCCATCGGCTCGTCC
pGG08 REV	AGTCGACCTGCAGGCATGCACACAGATGTAATTGCTGGTCACG
pGG09 FWD	ATACGACTCACTATAGGGAATCAGCCATCGGCTCGTCC
pGG09 REV	AGTCGACCTGCAGGCATGCAATCCCCGAAGATACACCGTAG
pGG10 FWD	ATACGACTCACTATAGGGAACCGGCGAACCCATCGACA
pGG10 REV	AGTCGACCTGCAGGCATGCATCATGCGGTCGTCCCAGTC
pGG21 FWD	ATACGACTCACTATAGGGAAGCCGGGAGCATACTTCCG
pGG21 REV	AGTCGACCTGCAGGCATGCAAACCAAAACAGGCCCCCTC
pGG22 FWD	ATACGACTCACTATAGGGAATTCCTCCTTTCCGCCTGC
pGG22 REV	AGTCGACCTGCAGGCATGCATCATTGTGGCGGTACCAC
pGG24 FWD	ATGACGCAGCCTCTGCGG
pGG24 REV	TTGTTCGCATCCCCCTTGAAC
pGG36 FWD	GATTCGCCGCCCGAAATCACGACCGATGAACCTGATCAAGATGAC
pGG36 REV	GCGTTTAAACCTGCAGGCACTCATCTGGGCACTCCGGC
pGG37 FWD	GATTCGCCGCCCGAAATCACTAAAAGGCGGTGGCTGAAC
pGG37 REV	GCGTTTAAACCTGCAGGCACCTACTTCATGAGGCCGAG
pGG38 FWD	GATTCGCCGCCCGAAATCACGACGAGAGGAACCACCTAAAATG
pGG38 REV	GCGTTTAAACCTGCAGGCACTTAGGCGCAAACCTTACG
LilD136_ptnd_Fwd	GATTCGCCGCCCGAAATCACCGACGAGAGGAACCATCG
Wilder140_ptnd_Fwd	GATTCGCCGCCCGAAATCACCGACGAGAGGAACCACCG
Wilder_LilD_ptnd_Rev	GCGTTTAAACCTGCAGGCACCTAGGCGCAAACCTTACG
