# Supplementary material for: More Evidence of Collusion: a New Prophage-Mediated Viral Defense System Encoded by Mycobacteriophage Sbash
Source: mBio. 2019 Mar 19;10(2):e00196-19. doi: 10.1128/mBio.00196-19 (PMC6426596; doi:10.1128/mBio.00196-19)
Supplement: TABLE S2 [file mBio.00196-19-st002.pdf]

Table S2. Plasmids constructed in this study

Plasmid	Vector	Phage Gene(s)	Phage Coordinates
pGG01	pJV39	Sbash *30-31*	26,724 – 28,594
pGG02	pJV39	Sbash *37-39*	31,562 – 33,214
pGG03	pGH1000	Sbash *32-34*	28,508 – 29,501
pGG04	pGH1000	Sbash *35-36*	29,232 – 31,621
pGG05	pGH1000	Sbash *30-31*	26,713 – 28,600
pGG06	pGH1000	Sbash *37-39*	31,544 – 33,259
pGG07	pGH1000	Sbash *43*	35,114 – 36,076
pGG08	pJV39	Sbash *41*-42	34,455 – 35,174
pGG09	pJV39	Sbash *41-43*	34,455 – 36,076
pGG10	pJV39	Sbash *30*	26,713 – 28,051
pGG12	pGH1000	Sbash *31* R70A	26,713 – 28,600
pGG13	pGH1000	Sbash *31* D73A	26,713 – 28,600
pGG21	pGH1000	Sbash *39*	32,520 – 33,259
pGG22	pGH1000	Sbash *35*	29,232 – 30,830
pGG24	pGG05	Sbash *31*	28,011 – 28,600
pGG36	pCCK39	Sbash *31*	27,991 – 28,508
pGG37	pCCK39	Crossroads *132*	68,249 – 69,120
pGG38	pCCK39	Crossroads *141*	73,007 – 74,268
pGG39	pCCK39	Crossroads DEM phgg399 *141*	73,007 – 74,268
pGG40	pCCK39	Crossroads DEM phgg364 *141*	73,007 – 74,262
pKSW01	pCCK39	Crossroads DEM 1_2A *141*	73,007 – 74,268
pKSW02	pCCK39	Crossroads DEM 4_1A *141*	73,007 – 74,268
pKSW03	pCCK39	Crossroads DEM phgg409 *141*	73,007 – 74,268
pKSW04	pCCK39	LilDestine *136*	71,926 – 73,187
pKSW05	pCCK39	Wilder *140*	72,610 – 73,871
pKSW06	pCCK38	Crossroads *132*	68,249 – 69,120
