# Supplementary material for: More Evidence of Collusion: a New Prophage-Mediated Viral Defense System Encoded by Mycobacteriophage Sbash
Source: mBio. 2019 Mar 19;10(2):e00196-19. doi: 10.1128/mBio.00196-19 (PMC6426596; doi:10.1128/mBio.00196-19)
Supplement: TABLE S3 [file mBio.00196-19-st003.pdf]

Table S3. DNA substrates and primers using for phage engineering

Name	G-block coordinates[1]	Primers[2]	Deletion Coordinates[3]
SbashΔ30	26,704 – 26,953;<br>28,001 – 28,250	Sbash30KO FWD:<br>GATGACTGACCGGCGAACCCATC<br><br>Sbash30KO REV:<br>CCACGGCTCGCTACCGCC	26,954 – 28,000
SbashΔ31	27,812 – 28,061;<br>28,488 – 28,702	Sbash31KO FWD:<br>ACGATGACGATCGTGATGGATGGCG<br><br>Sbash31KO REV:<br>ACAGTGCGCGGCTGGACC	28,062 – 28,487
SbashΔ34	28,865 – 29,108;<br>29,223 – 29,472	Sbash34KO FWD:<br>GCGACGCGGTTGCCGATCT<br><br>Sbash34KO REV:<br>CTACCGGGATACGCTACCGCCG	29,109 – 29,222
SbashΔ35	29,273 – 29,522;<br>30, 810 – 31,059	Sbash35KO FWD:<br>GACTTTACCCTAAGCAAGGGTGTAGTTC<br><br>Sbash35KO REV:<br>GATGTGATCGCGGCCTCGAG	29,523 – 30,809
SbashΔ37	31,380 – 31,629;<br>31,765 – 32,014	Sbash37KO FWD:<br>ACATCCGCGACGGAATCTCGTT<br><br>Sbash37KO REV:<br>GGGTGATGACTACTGCGCGC	31,630 – 31,764
SbashΔ38	31,586 – 31,835;<br>32,499 – 32,748	Sbash38KO FWD:<br>CGAAAGGGTGTAGTCGTACTCAAATCTCCT<br><br>Sbash38KO REV:<br>ACCACCATCGGCATCATCCAGC	31,836 – 32,498
SbashΔ39	32,352 – 32,601;<br>32,959 – 33,208	Sbash39KO FWD:<br>CGCGATGGACGTGATCCAGAACG<br><br>Sbash39KO REV:<br>GATTCGAACCTGCGTAGGCGTAAGC	32,602-32,958
CrossroadsΔ132	68,020 – 68,269;<br>69,080 – 69,329	Crossroads132KO FWD:<br>CCAGTCTGGCGGCTGGTCGG<br><br>Crossroads132KO REV:<br>TCAACCCTGGCGCTCGCGG	68,270 – 69,079
CrossroadsΔ141	72,778 – 73,027;<br>74,228 – 74,477	Crossroads141KO FWD:<br>CCGTGGTTCGTGACCTTCATTTTCGT<br><br>Crossroads141KO REV:<br>CGCTGGCTTAAGCGTCGCTAGT	73,028 – 74,227
CrossroadsΔ140-141	71,743-71,942;<br>74,249-74,448	Crossroads140_141KO_Fwd_V2:<br>GCCATGCGACGGGGACCG<br><br>Crossroads140_141KO_Rev_V2:<br>GATACCGACACATAACGAGACCTAAGCG	71,943-74,248
CrossroadsΔ139-141	70,510-70,766;<br>74,273-74,515	CrossroadsKO139_141_Fwd:<br>CGTCGTCTGCCTCGGTGTC<br><br>CrossroadsKO139_141_Rev:<br>GACACGAACCAAGCAGCTC	70,767-74,272

[1]Each G-block was designed to include ~250bp upstream and downstream of the deletion end points, and the coordinates for each are shown.
[2]Primers are designed to anneal to the flanks of the G-block substrate and were used to amplify the substrate prior to co-electroporation with phage genomic DNA.
[3]Deletion coordinates correspond to the wild-type phage sequence
